# Determination of Base Binding Strength and Base Stacking Interaction of DNA Duplex Using Atomic Force Microscope

**DOI:** 10.1038/srep09143

**Published:** 2015-03-16

**Authors:** Tian-biao Zhang, Chang-lin Zhang, Zai-li Dong, Yi-fu Guan

**Affiliations:** 1 Department of Biochemistry and Molecular Biology, China Medical University, Shenyang, China. 110001; 2 State Key Laboratory of Robotics, Shenyang Institute of Automatics, Chinese Academy of Sciences, Shenyang, China. 110016

## Abstract

As one of the most crucial properties of DNA, the structural stability and the mechanical strength are attracting a great attention. Here, we take advantage of high force resolution and high special resolution of Atom Force Microscope and investigate the mechanical force of DNA duplexes. To evaluate the base pair hydrogen bond strength and base stacking force in DNA strands, we designed two modes (unzipping and stretching) for the measurement rupture forces. Employing k-means clustering algorithm, the ruptured force are clustered and the mean values are estimated. We assessed the influence of experimental parameters and performed the force evaluation for DNA duplexes of pure dG/dC and dA/dT base pairs. The base binding strength of single dG/dC and single dA/dT were estimated to be 20.0 ± 0.2 pN and 14.0 ± 0.3 pN, respectively, and the base stacking interaction was estimated to be 2.0 ± 0.1 pN. Our results provide valuable information about the quantitative evaluation of the mechanical properties of the DNA duplexes.

DNA double helical structure plays an important role in life science because it serves not only as the genetic information carrier but also as the structural basis for biological processes of DNA replication and transcription. These helical structures are stabilized by the hydrogen bonds of the base pairs as well as the stacking interactions between two adjacent overlapped base pairs. A great effort has been devoted to explore the accurate information about the binding force between base pairs^1^,^2^,^3^,^4^. However, traditional techniques are heavily suffered from high cost, low efficiency and low force resolution.

With the advent of atomic force microscope (AFM), unprecedentedly high force resolution at piconewton (pN) could be achieved. In the past two decades, AFM has proved to be an outstanding means for evaluation of the mechanical properties of DNA^5^,^6^,^7^,^8^,^9^,^10^,^11^,^12^,^13^. Lee *et al*. conducted a pioneering work of measuring the binding force of DNA duplex using AFM^6^. They designed a clever experiment in which a DNA sequence (ACTG)_5_ could form duplexes of defined lengths (20, 16 and 12 bp) and the adhesive force were centered at 1.52, 1.11 and 0.82 nN, representing the rupture force of interchain interactions between a single pair of duplexes, respectively^6^,^13^. DNA duplex stretching experiments were then widely adopted to test the pN resolution force upon base pair rupture^6^,^10^,^11^,^12^,^14^. In some early experiments, the impact of electrostatic screening effect on the duplex lengths^1^ and the influence of hydrodynamic coupling between DNA and the fluid was investigated^14^. Many groups showed interest in the twist stored within the double helix^15^,^16^,^17^. Furthermore, some researchers have demonstrated the correlation between the average dG/dC content of DNA duplexes and the measured base pair rupture force strength^15^,^18^,^19^. AFM measurement could even identify base mutation, which highlighted the potential application in DNA sequencing and diagnosis of genetic diseases^20^,^21^.

In spite of the great success achieved in AFM characterization of mechanical properties of DNA duplexes, previous studies using AFM have demonstrated some inconsistent results. The rupture forces of DNA duplexes were reported in a relatively wide range^5^,^8^,^9^,^10^,^11^,^12^,^20^,^22^,^23^. It could be as large as 300 pN^23^ and as small as 10 pN as well^18^. These discrepancies in the rupture force of DNA duplexes indeed created confusion. In addition, carefully reviewing the previously published data found that the measured binding strengths in different experiments have never been compared, and most of the previous studies have been focused only on the characterization of the hydrogen bonds which stabilize the double strand DNA laterally. It has been known that the base stacking interactions contribute also to the DNA stabilization^7^, and the base stacking interactions demonstrated the sequence-dependent property^24^. Unfortunately, the base stacking interaction has been much less investigated so far.

In this study, we designed two modes of force field measurement. As shown in Figure 1, in the unzipping mode, the 5′-end of one strand is covalently linked to the cantilever tip and the 3′-end of the complementary strand is attached to the glass slide. After a duplex is formed, moving the cantilever tip upward will unwind the duplex. Thus, the base pairs will be separated one by one, and the measured rupture force represents the hydrogen bond strength of single base pair. In contrast, in the stretching mode, the 3′-end of two oligonucleotides are covalently tethered to the cantilever tip and the glass slide, respectively. Thus, as the cantilever tip moves upward, two strands of this duplex will be separated by pulling apart in opposite directions. Thus, the measured rupture force will include the hydrogen bond interaction of base pairs and base sacking interaction between adjacent base pairs. Comparison of these two types of rupture forces enables us to distinguish the hydrogen bond strength as well as the base stacking interaction. In order to reveal the rupture force more precisely, we initially characterized the dependence of these rupture forces on experiment parameters, including Na^+^ concentration, cantilever holding time, loading rate and DNA chain length.

## Methods

### Chemicals and oligonucleotides

All chemicals used in this study were purchased from Fluka Chemical Corporation (Milwaukee, WI, USA). The standard gold AFM cantilevers (No. CSC38) were purchased from MikroMasch Company (Soquel, CA 95073, USA). The spring constant of the AFM cantilevers used in this experiment was 30 pN/nm and each cantilever was calibrated with the thermal tune module (Digital Instruments Inc.). The aldehyde-modified glass slides were purchased from CapitalBio Corporation (Beijing, China). All oligonucleotides at the HPLC purity were synthesized by GenScript Corporation (Shanghai, China).

### Surface modification and oligonucleotide immobilization

To immobilize these oligonucleotides on the cantilever tip and the aldehyde-modified glass slides, these oligonucleotides have been modified at either the 5′-end or the 3′-end with -SH or -NH_2_ group, respectively (Table 1). Then, these oligonucleotides are immobilized on the tip of the AFM cantilever or the glass slide. To maintain the flexibility of the oligonucleotides, a (-CH_2_-)_6_ linker has been inserted between the nucleotide sequence and the functional group at the terminal.

The glass slides were sonicated in an ethanol bath for 20 min, and dried in a stream of argon gas. Immediately before use, the oligonucleotides SH1-SH4 with a 5′-SH (or 3′-SH) group were prepared at a final concentration of 10 μM in TE buffer (pH 7.4). The AFM cantilever tip was immersed in the oligonucleotide solution at room temperature for 30 min, and then rinsed with PBS buffer (pH 7.3) thoroughly. Oligonucleotides with a -NH_2_ group at the 3′-end were prepared at a final concentration of 5 μM in TE buffer (pH 7.4). 10 μl of the oligonucleotide solution was spread on the glass slide surface, stored in a humid chamber at room temperature for 24 h, and then dried at 80°C for 1 h. After rinsing with PBS buffer (pH 7.3), the slides were ready for experiments. Immobilization of oligonucleotides is the same for both unzipping and stretching measurement modes.

### AFM setup and hydrogen bond strength measurements

The measurements were conducted on AFM (Dimension 3100, Digital Instruments Inc, Santa Barbara, CA, USA) with the Nanoscope IV controller. All the experiments were performed at room temperature. Before measurement, the spring constant of each AFM cantilever was calibrated using the Hutter's method to ensure the data accuracy^14^. Cantilever deflections versus piezo displacement were recorded by optical lever detection with Nanoscope software (V6.13). Force measurements were performed with a 100–200 nm z-axis piezo. The cantilever deflections with respect to the z-piezo displacement were transformed into force strength using the cantilever sensitivity and the spring constant. Each force curve consisted of 512 data points. In each measurement, AFM tip was emerged into the TE buffer in a round petri dish, and kept in the solution for certain periods before the force curve measurement. After selecting proper parameters, the force curves are collected in an array of 6 points × 6 points with 100 nm intervals.

**Figure 2:**
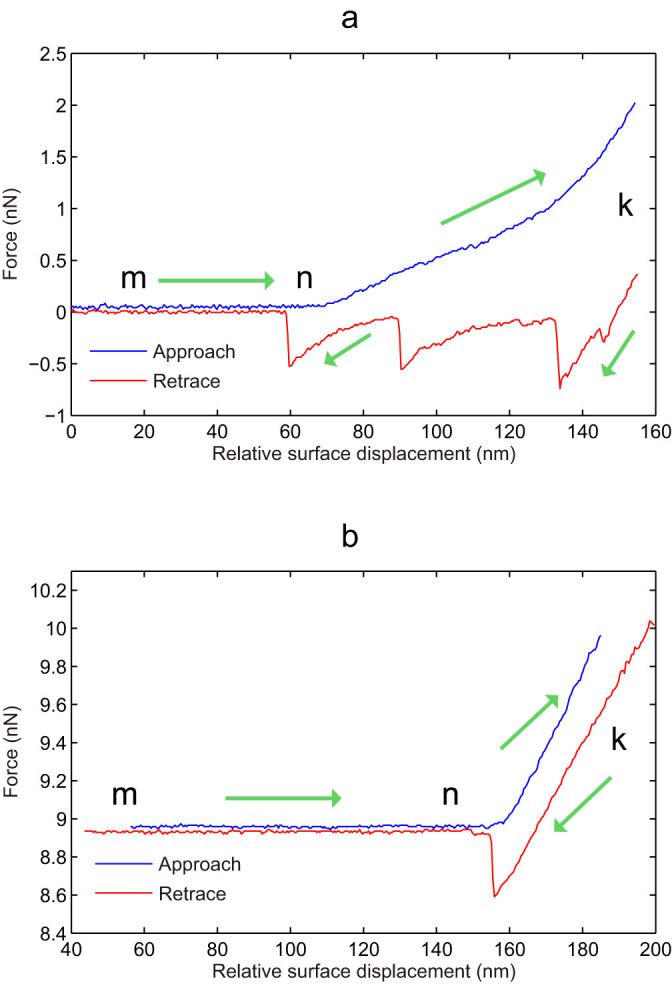
Force curves showing the rupture force versus relative surface displacement of the cantilever tip. The blue line and the red line represent the approaching and retracing processes, respectively. (a): sequential rupture of three DNA duplexes; (b): one rupture of single DNA duplex.

The collected force curves, that is, the relative force strength versus the AFM cantilever displacement, are shown in Figure 2. From point “m” to point “n” on the blue line represents the tip approaching toward the surface. When the AFM tip touches the surface (point “n”), the cantilever starts to bend, and as the z-piezo running down continually until point “k”, which is determined by the contact force threshold (pre-set by users) between the tip and the surface. Maintaining the tip at this position for a certain period allows the duplex formation between one oligonucleotide immobilized on the AFM cantilever tip and one complementary strand immobilized on the glass slide surface.

The red line represents a typical tip retracting process, that is, the tip leaves from the surface. Thus, the duplexes undergo strand separation process. As shown in Figure 2, the downward peaks indicate the duplex rupture. It should be noted that the number of DNA duplex formed between the tip and the glass slid cannot be guaranteed to be uniform. As indicated in Figure 2a, several peaks in the retracting curves indicate the breakages of several DNA duplexes in the unzipping mode measurement, while a single big rupture peak was usually observed for DNA duplex in the stretching mode measurement (Figure 2b).

The glass slide surface was modified with a layer of single-stranded oligonucleotide, whereas the AFM tip is tethered with a complementary oligonucleotide. Upon retraction, DNA duplexes were stretched between the glass surface and the tip by applying a force of 0.01 ~ 2.00 nN.

### Data processing

In general, there are several rupture peaks in each force curve (Figure 2). We extracted these force values using the Nanoscope software (Ver.613). All the collected data were analyzed using homemade software designed based on the principle of unsupervised k-means clustering algorithm.

The k-means clustering algorithm is to partition the data into a special cluster with the nearest mean value. The process can be accomplished as following. First, based on the reported results, we chose six k values as the mass centers from all the collected data. Then, the least square of the distances of every force value to each mass center were calculated, and a new mass center was selected for each k value, and the least square calculations were repeated. This clustering process will be performed iteratively until the value of a mass center reached stable. The mass center is considered as the rupture forces of duplexes. The flow chart of the k-means clustering algorithm is provided in Figure S2 in Supplementary Information. Division of these duplex rupture forces by the base pair number in DNA duplexes will lead to the binding strength of single base pair. Furthermore, the single base pair rupture force for both the unzipping mode and the stretching mode could be determined, and the base stacking force would be obtained by comparison of rupture forces determined from these two modes.

## Results and Discussion

### Effects of NaCl concentration and holding time

Formation of a stable DNA duplex is essential to the acquisition of the highly reproducible force curves for determining hydrogen bond strengths and base stacking interactions. Ionic interactions are critical to the stabilization of the DNA duplex structures since the cations can neutralize the negative charges on the phosphate groups of the DNA backbone^25^,^26^. In this study, the ionic effect was examined first and the optimal ionic concentration was used for the following experiments. Based on the common physiological knowledge and previous literature^25^, three NaCl concentrations of 0, 50 and 100 mM were tested.

The holding time is defined as the duration that the oligonucleotide-tethered cantilever tip is kept in contact with the glass surface for DNA duplex formation (point “k” of force curve in Figure 2). This contact allows the oligonucleotide immobilized on the glass surface to hybrid with its complementary oligonucleotide tethered on the cantilever tip. Three different holding times of 0, 10 and 40 seconds were selected. Thus, there are nine combinations as shown in Table 2. In each situation, 100 force curves were collected and only the force curves with a series of valid rupture peaks were considered for the data processing.

The experimental results demonstrated the effect of Na^+^ cation on hydrogen bond formation. Only very noisy curves were obtained when NaCl was absent in the solution no matter how long the holding time was (data not shown). As the NaCl concentration was increased from 0 mM to 50 mM, the rupture forces increased significantly from 14 pN to 56 pN (Table 2). Further increase of the NaCl concentration was able to enhance the force strength moderately (from 56 pN to 70 pN). These data are consistent with the previous report where the DNA duplexes were considerably destabilized under low salt conditions (10 mM NaCl), while high ionic strength buffers (1 M NaCl) stabilized the duplex conformation^23^,^27^,^28^,^29^. It has been observed that when the dsDNA molecules in aqueous buffer underwent a highly cooperative transition into an extended stable form, the ionic strength of the medium plays an important role^30^.

In general, hydrogen bonds could be formed within a few milliseconds, however, forming a stable DNA duplex might take a longer time, providing more opportunities for two complementary strands to undergo repeated association-dissociation and eventually wind together to form a stable duplex. Zero holding time means that the AFM tip begins to retract immediately once it touches the glass surface. As displayed in Table 2, when the holding time changed from 0 second to 10 seconds, the rupture forces increased from 27 pN to 56 pN at 50 mM NaCl concentration, and from 37 pN to 70 pN at 100 mM NaCl concentration. When the holding time was extended to 40 seconds, we observed a small decline of rupture force (Table 2), meaning that the longer holding time is not beneficial to the duplex formation. It is could be due probably to the AFM system drafting. Thus, the holding time of 10 seconds was chosen in the following experiments. At the same time, we found that there were more rupture peaks on the force curves collected at 100 mM NaCl than those at 50 mM NaCl, indicating that Na^+^ could stabilize the DNA base pair. Furthermore, the intervals between two cluster centers under the condition of 100 mM NaCl are very nearly the same.

Several rupture peaks in the later region of force curves were noticed. However, their magnitudes fell in the region of 0.8 nN–1.0 nN. These values seemed to be too large to be the true rupture forces. This phenomenon has been reported in other previous studies^13^. Therefore, we speculated that these peaks could be due primarily to the non-specific interaction between the cantilever tip and the glass surface rather than the hydrogen bond breakage, thus, we excluded them from the data analysis.

### Effect of loading rate in different modes

To optimize the data acquisition conditions, we utilized different approaching and retracting velocities. The loading rate at the unit of nm/s could be converted to the unit of pN/s by multiplying the spring constant of the cantilever (10 pN/nm). In this trial, we used 14 bp oligonucleotide and applied both stretching mode and unzipping mode to estimate the hydrogen bond strength.

**Figure 3:**
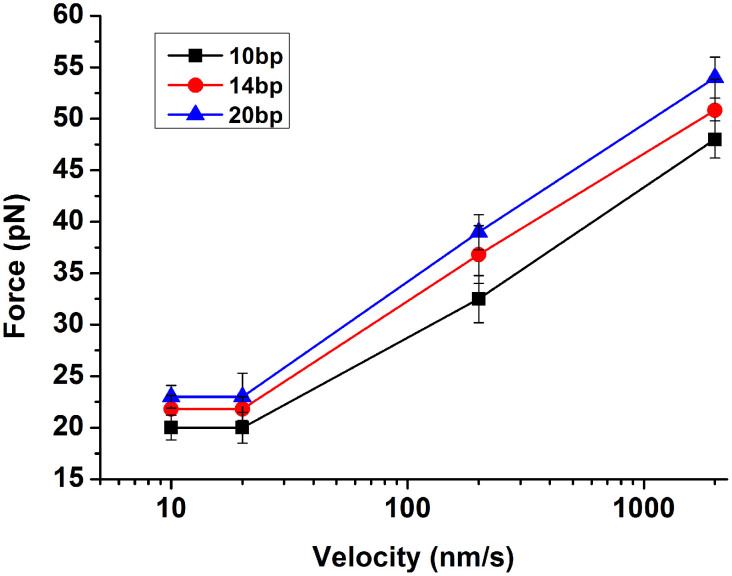
Dependence of rupture force on the loading rates for different lengths of dG/dC base pair using the stretching mode.

It is interesting to notice that the unzipping mode (separating the two strands of the duplex by unwinding from one end of the duplex) always showed very constant values of the rupture force for the dG/dC pair and dA/dT pair regardless of the loading rate (Table 3). The rupture force was about 20.0 ± 0.2 pN for one dG/dC base pair and 14.0 ± 0.3 pN for one dA/dT base pair, respectively. However, the rupture force increased as the loading rate increased when the stretching mode was applied. Both dG/dC base pair and dA/dT base pair displayed the same trend (Table 3 and Figure 3). We observed similar results for shorter (10-bp) and longer oligonucleotides (20-bp). Previous studies have also exhibited a pronounced force-loading-rate dependence^23^.

It has been observed in previous studies that the rupture force has a relationship with the loading rate. Depending on the loading rates in the range of 16–4,000 pN/s, the binding forces of a duplex varied from 20 pN to 50 pN. These binding forces were found to scale with the logarithm of the loading rate, which was interpreted in terms of a single energy barrier along the mechanical separation pathway^8^. In the stretching mode, with the increased loading rate, separation of the complementary strands proceeded in a stepwise manner. B-form DNA was stretched, then structurally transformed to a stable form of DNA approximately twice the length of the B-form, and finally separated into single-stranded oligonucleotides. These data indicated that a direct measurement of the rupture forces involved anelastic deform of a double-stranded DNA^12^. The effects of different measurement modes on the experimental data could be interpreted as following. Unzipping the duplex is equivalent to separating base pairs progressively one by one, thus, retracting velocity has no effect on the rupture forces. In contrast, stretching the duplex from both ends is just to pull the two strands apart from each end, thus, higher retracing velocity could create a beneficial to the breakage of the duplexes.

### Determination of hydrogen bond force and base stacking interaction

To ensure the data reliability, we utilized fully complementary oligonucleotide duplexes having 10, 14 and 20 dG/dC and dA/dT base pairs, respectively. We set the NaCl concentration at 100 mM, holding time at 10 seconds and loading rate at 20 nm/s. Taking 14-dG/dC duplex and the unzipping mode as an example, we collected forces curves from an array of 6 points × 6 points on the slide surface, and repeated the collections at each point several times.

**Figure 4:**
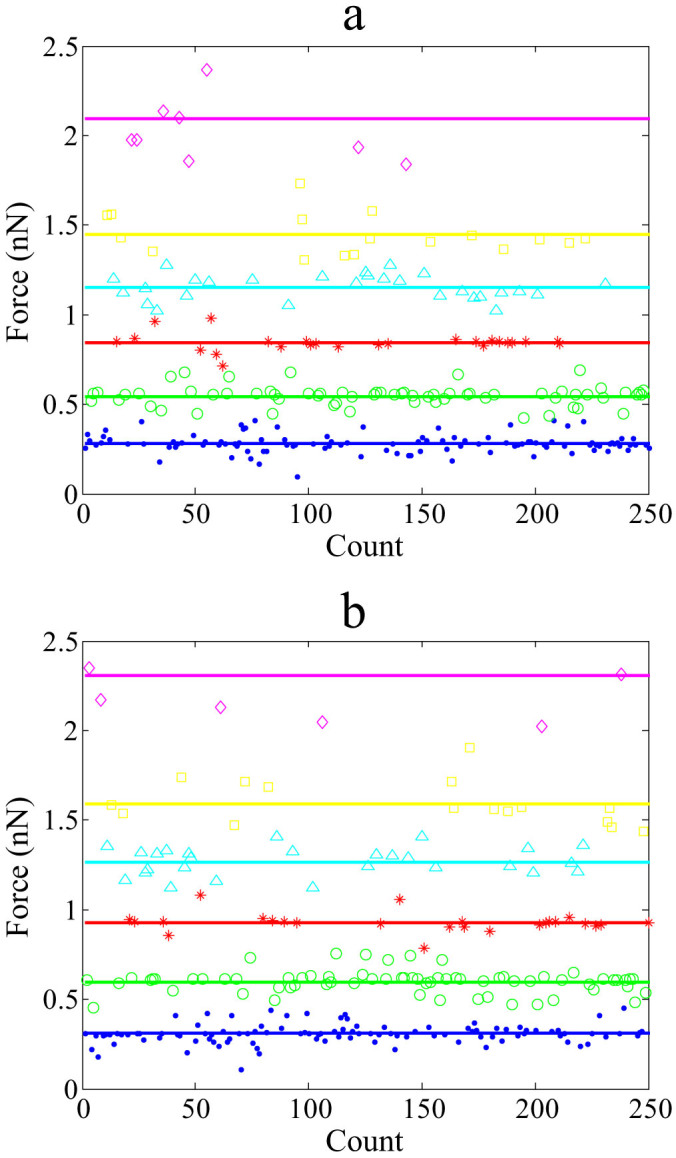
The clustered rupture force using the algorithm of k-means clustering. All the rupture forces were obtained under the condition of 100 mM NaCl condition and 10 s holding time. (a) a duplex of 14-dG/dC base pairs using the unzipping mode; (b) a duplex of 14-dG/dC base pairs using the stretching mode.

We designed the data processing software using MATLAB. Once the raw data were input, the data were clustered, and the mean values of the clustered numbers were calculated. Each diagram in Figure 4 shows 6 straight horizontal lines in different color, each representing one mean value of the most popularized data points. The relevant force value of each center is displayed in Table 4,5,6,7. Using 14-dG/dC duplex and unzipping mode, the intervals between two adjacent lines are 0.2837, 0.2604, 0.3001, 0.3077 and 0.2948 nN from bottom to top, respectively, fluctuating around 0.3 nN. The bottom blue line indicates one duplex strand rupture, and the green line represents two duplexes rupture (Figure 4(a)). The next three lines show the same tendency; but the top line exhibits a large fluctuation which could be due to the interference with the nonspecific adhesion force.

The percentages of each clustered force value in all data points were also summarized in Table 4,5,6,7. In the case of the 14-dG/dC under the condition of 100 mM NaCl concentration and 10 s holding time (Table 4), the data points around the blue line (0.2837 nN) account for 41.96% of the total data points, and the data points around the green line (0.5441 nN) account for 27.06%. Thus, we conclude that the events of one duplex rupture and two duplex rupture are predominant in the experiments. Furthermore, we estimated the base pair strength from the values in Table 4, division of 0.282 nN by the base number 14 yielded one dG/dC base pair strength of about 20.1 pN. The same procedure was also applied to the duplexes of 10-bp and 20-bp, and the calculated values for one base pair exhibit a high consistence (20.0 pN and 20.1 pN). Through averaging of results for the three situations of 10 bp, 14 bp and 20 bp, the binding strengths of dG/dC and dA/dT base pair were determined to be 20.0 ± 0.2 pN and 14.0 ± 0.3 pN, respectively. It has been well known that dG/dC has three hydrogen bonds and dA/dT has only two, the calculated hydrogen bond strength are reasonable.

We have compared our data with other previous investigations. Using the AFM tip coated with a type of nucleotide (A or C) and a surface coated with A, T, G or C, a separation force of 54 pN was obtained for a single AT base pair^5^. In the experiment of Lee *et al*., they obtained 1520, 1110 and 820 pN for duplexes of 20, 16 and 12 bp, respectively, which were equivalent to 76, 69 and 68 pN per base pair^6^. These numbers seem to be higher than the values given in many other experiments. Careful examination showed that it is due probably to a higher loading rate used for data collection and might include a strong coupling between bases because of the shearing motion. Experiments using poly (dG-dC) and poly (dA-dT) sequences and stretching mode revealed a close correlation between the mechanical strength of these sequences with base pairing. Other results showed that, depending on the loading rate, the base pairing strength for poly(dG-dC) DNA was about 300 pN, whereas it was only 35 pN for poly(dA-dT) DNA^23^. Strunz *et al*. has investigated DNA duplexes with 10, 20, and 30 base pairs with loading rates in the range of 16–4,000 pN/s. By pulling apart these duplexes at the opposite 5′-ends, the binding forces of single duplexes were measured in the range of 20 to 50 pN depending on the loading rate and duplex length^8^. Rief's study of poly(dG-dC) and poly(dA-dT) using the unzipping mode has derived the following data: the dG-dC hydrogen bond force was 20.0 ± 3.0 pN, and the dA-dT was (9.0 ± 3.0) pN^10^. Similarly, Essevaz-Roulet's work has determined a typical binding force of bacteriophage λ-DNA in the range of 10–15 pN for an AT base pair^18^. Interesting report has also be identified that under a longitudinal stress of approximately 65 pN, DNA duplexes in aqueous buffer underwent a highly cooperative transition into a stable form with 5.8 angstroms rise per base pair, that is, 70% longer than B form dsDNA, while when single-stranded DNA was stretched, it gave a persistence length of 7.5 angstroms, the measured stretch modulus was about 800 pN. The authors speculated that the overstretched form may play a significant role in the DNA recombination^30^.

Stacking interaction of the base pairs is another essential force component responsible for the DNA duplex stabilization. However, the actual strength of the stacking interaction has been rarely reported. Sattin *et al.* used a (GTCA)_5_ oligonucleotide and a complementary oligonucleotide as the model to investigate the absolute force cost and relative force cost of unstacking an AT base pair. They found that force cost decreased as the number of H-bonds in the double helix decreased, from 5.5 pN at 45 H-bonds to 0.5 pN at 23 H-bonds. In contrast, unstacking AC/TG base pairs would cost more than unstacking the AT base pair. In general, this force cost increased as the number of total H-bonds decreased, starting at 5 pN for 43 H-bonds and reaching a maximum of 8 pN at 29 H-bonds. Thus, they concluded that unstacking a single AT base pair did not create much impact on destabilizing a double helix^7^.

As mentioned, when comparing the binding strength of base pairs obtained using the unzipping mode and stretching mode, we could derive some information about the base stacking interaction. As shown in Table 3, when the loading rates were below 20 nm/s, the base stacking forces for dG/dC as well as for dA/dT base pair were about 2 pN. However, when the loading rate was increased to 200 nm/s, the base stacking forces were 19 pN for dG/dC and 17 pN for dA/dT base pair, respectively, and further increase of the loading rate to 2000 nm/s, the stacking forces were estimated to be 29 and 25 for dG/dC and dA/dT base pair, respectively. It is clear that the loading rate has a profound impact on the force measurement.

## Conclusion

In this study, we have evaluated binding strength of DNA duplexes of different lengths using AFM. By employing the unzipping mode and the stretching mode, the binding strength of dG/dC and dA/dT base pairs have been determined to be 20.0 ± 0.2 pN and 14.0 ± 0.3 pN, respectively. In comparison with the previously reported data, the data accuracy has been greatly improved in this work. Thus, these data could represent the native nature of these DNA duplexes. Furthermore, base stacking interactions between two adjacent base pairs have been also estimated to be about 2 pN, which is of prodigious significance since it has been much less investigated previously. Experimental results also proved that the NaCl concentration is a key factor for the DNA duplex formation. Hydrogen bond cannot be formed without Na^+^. In addition, a holding time of at least several seconds is required to form stable DNA duplexes. The results and the conclusions in this study have forwarded valuable information about the DNA duplex formation and the application potentials in mismatch detection and medical diagnosis in future.

## Author Contributions

T.Z., C.Z., Z.D. and Y.G. designed and performed the experiments, and analyzed data. T.Z. and Y.G. drafted the manuscript.

## Supplementary Material

Supplementary Information Supporting Information

## Figures and Tables

**Figure 1:**
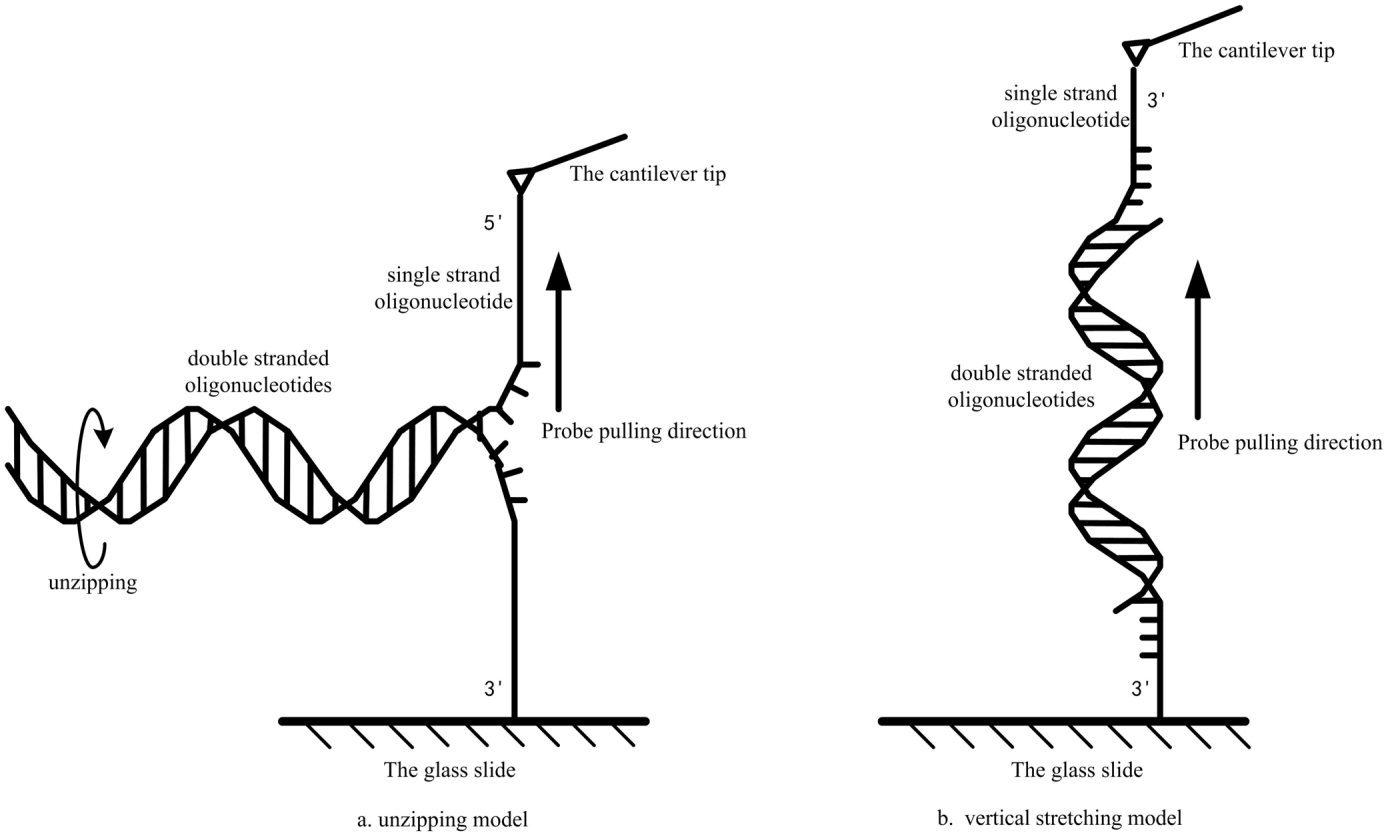
Schematic diagram of the unzipping mode and the stretching mode used for measurement of the binding strength of the base pairs.

**Table 1:** Oligonucleotides used for measurement of the binding strength of dA/dT and dG/dC base paris

	sequences	notation
SH1	5′-SH-(CH_2_)_6_-(G)_30_-3′	Cantilever tip
SH2	3′-SH-(CH_2_)_6_-(G)_30_-5′	Cantilever tip
dC10	3′-NH_2_-(CH_2_)_6_-(C)_10_-5′	Glass slide
dC14	3′-NH_2_-(CH_2_)_6_-(C)_14_-5′	Glass slide
dC20	3′-NH_2_-(CH_2_)_6_-(C)_20_-5′	Glass slide
SH3	5′-SH-(CH_2_)_6_-(A)_30_-3′	Cantilever tip
SH4	3′-SH-(CH_2_)_6_-(A)_30_-5′	Cantilever tip
dT10	3′-NH_2_-(CH_2_)_6_-(T)_10_-5′	Glass slide
dT14	3′-NH_2_-(CH_2_)_6_-(T)_14_-5′	Glass slide
dT20	3′-NH_2_-(CH_2_)_6_-(T)_20_-5′	Glass slide

**Table 2:** Dependence of the rupture force on ionic concentration and holding time

NaCl concentration (mM)	0			50			100		
Holding time (s)	0	10	40	0	10	40	0	10	40
Total curves	60	60	61	74	76	49	72	88	68
Rupture force (pN)	3	14	15	27	56	40	37	70	62

Data were obtained in solution with 14-bp dG/dC duplex using stretching mode.

**Table 3:** Binding strength (pN) obtained under different loading rate and two different modes for dG/dC and dA/dT base pairs

	G-C		A-T	
loading rate (nm/s)	stretching mode	unzipping mode	stretching mode	unzipping mode
10	22 ± 1.25	19 ± 1.05	16 ± 1.37	14 ± 1.76
20	22 ± 1.34	20 ± 1.54	16 ± 1.64	14 ± 1.64
200	38 ± 2.46	19 ± 1.26	30 ± 2.54	13 ± 1.09
2000	50 ± 2.52	21 ± 1.38	40 ± 2.96	15 ± 1.62

Data were obtained with 14-bp dG/dC duplex.

**Table 4:** Binding strength obtained using the unzipping mode for dG/dC base pair of different lengths

Length of nucleotide chain(bp)	Hydrogen bond force (nN) Cluster centers distribution (%)					
10	0.2029 (32.72)	0.3990 (28.87)	0.6056 (5.80)	0.8041 (13.49)	1.0031 (11.57)	1.2293 (7.73)
14	0.2837 (41.96)	0.5441 (27.06)	0.8442 (9.81)	1.1519 (10.98)	1.4467 (6.67)	2.0958 (3.53)
20	0.4019 (37.52)	0.8227 (28.95)	1.2124 (17.78)	1.5794 (8.95)	2.0370 (3.72)	2.4636 (3.14)

Data were obtained under condition of 100 mM NaCl concentration and 10 s holding time. Data in parentheses are the percentage of the binding strengths in the whole clustering data.

**Table 5:** Binding strength obtained using the stretching mode for dG/dC base pair of different lengths

Length of nucleotide chain(bp)	Hydrogen bond force (nN) Cluster centers distribution (%)					
10	0.2231 (40.39)	0.4389 (28.24)	0.6661 (9.81)	0.8845 (10.98)	1.1034 (7.45)	1.3522 (3.14)
14	0.2846 (40.86)	0.5985 (29.72)	0.9286 (10.85)	1.2671 (9.79)	1.5913 (5.52)	2.3054 (3.36)
20	0.4458 (39.12)	0.8550 (28.38)	1.3266 (16.48)	1.8101 (9.37)	2.2733 (3.42)	2.9147 (3.26)

**Table 6:** Binding strength obtained using the unzipping mode for the dA/dT base pair of different lengths

Length of nucleotide chain(bp)	Hydrogen bond force (nN) Cluster centers distribution (%)					
10	0.1418 (40.85)	0.2721 (28.49)	0.4221 (10.24)	0.5759 (9.94)	0.7233 (7.06)	1.0479 (3.45)
14	0.1962 (39.98)	0.3893 (29.46)	0.5939 (9.95)	0.7929 (10.49)	0.9822 (6.49)	1.2005 (3.64)
20	0.2840 (40.12)	0.5585 (28.84)	0.8478 (10.49)	1.1258 (10.18)	1.4044 (6.86)	1.7210 (3.56)

**Table 7:** Binding strength obtained using the stretching mode for the dA/dT base pair of different lengths

Length of nucleotide chain(bp)	Hydrogen bond force (nN) Cluster centers distribution (%)					
10	0.1621 (41.06)	0.3109 (30.26)	0.4824 (9.84)	0.6582 (9.36)	0.8267 (5.43)	1.1976 (4.06)
14	0.2246 (40.59)	0.4383 (29.46)	0.6889 (10.33)	0.9066 (9.56)	1.1650 (6.01)	1.3969 (4.06)
20	0.3242 (40.61)	0.6218 (29.38)	0.9648 (9.43)	1.3164 (10.51)	1.6533 (6.52)	2.3951 (3.57)
