# Supplementary material for: Determination of Base Binding Strength and Base Stacking Interaction of DNA Duplex Using Atomic Force Microscope
Source: Sci Rep. 2015 Mar 16;5:9143. doi: 10.1038/srep09143 (PMC4360479; doi:10.1038/srep09143)
Supplement: Supplementary Information — Supporting Information [file srep09143-s1.doc]

Supplementary Materials for

Determination of Base Binding Strength and Base Stacking Interaction of DNA Duplex Using Atomic Force Microscope

Tianbiao Zhang^1^, Changlin Zhang^2^, Zaili Dong^2^, Yifu Guan^1*^

^1^Department of Biochemistry and Molecular Biology, China Medical University, Shenyang, China, 110001

^2^State Key Laboratory of Robotics, Shenyang Institute of Automatics, Chinese Academy of Sciences, Shenyang, China, 110016

Figure S1 The clustered rupture force using the algorithm of k-means clustering. All the rupture forces were obtained under the condition of 100 mM NaCl condition and 10 sec holding time.

(a) 10 dA/dT base pair of the unzipping mode; (b) 10 dA/dT base pair of the stretching mode;

(c) 14 dA/dT base pair of the unzipping mode; (d) 14 dA/dT base pair of the stretching mode;

(e) 20 dA/dT base pair of the unzipping mode; (f) 20 dA/dT base pair of the stretching mode;

(g) 10 dG/dC base pair of the unzipping mode; (h) 10 dG/dC base pair of the stretching mode;

(i) 20 dG/dC base pair of the unzipping mode; (j) 20 dG/dC base pair of the stretching mode.

a b

c d

e f

g h

i j

Figure s2. Flow chart of the k-means clustering algorithm.
